# Collagen Crosslinking for Keratoconus: Cellular Signaling Mechanisms

**DOI:** 10.3390/biom13040696

**Published:** 2023-04-20

**Authors:** Dimitrios Karamichos, Sarah E. Nicholas, Asher Khan, Kamran M. Riaz

**Affiliations:** 1 North Texas Eye Research Institute, University of North Texas Health Science Center, 3500 Camp Bowie Blvd, IREB-505, Fort Worth, TX 76107, USA; 2 Department of Pharmaceutical Sciences, University of North Texas Health Science Center, 3500 Camp Bowie Blvd, Fort Worth, TX 76107, USA; 3 Department of Pharmacology and Neuroscience, University of North Texas Health Science Center, 3500 Camp Bowie Blvd, Fort Worth, TX 76107, USA; 4 Dean McGee Eye Institute, University of Oklahoma, 608 Stanton L Young Blvd, Oklahoma City, OK 73104, USA; 5 College of Medicine, University of Oklahoma, 800 Stanton L Young Blvd, Oklahoma City, OK 73117, USA

**Keywords:** collagen crosslinking, keratoconus, 3D self-assembled ECM model, primary corneal stromal fibroblast, Wnt signaling, corneal fibrosis, prolactin-induced protein (PIP), peroxisome proliferator-activated receptor-gamma coactivator 1 alpha (PCG-1), c-Src kinase, cyclin D1

## Abstract

Collagen crosslinking (CXL) is a widely used treatment to halt the progression of keratoconus (KC). Unfortunately, a significant number of patients with progressive KC will not qualify for CXL, including those with corneas thinner than 400 µm. The present study aimed to investigate the molecular effects of CXL using in vitro models, mirroring the normal, as well as thinner corneal stroma seen in KCs. Primary human corneal stromal cells were isolated from healthy (HCFs) and keratoconus (HKCs) donors. Cells were cultured and stimulated with stable Vitamin C resulting in 3D self-assembled extracellular matrix (ECM), cell-embedded, constructs. CXL was performed on (a) thin ECM with CXL performed at week 2 and (b) normal ECM with CXL performed at week 4. Constructs without CXL served as controls. All constructs were processed for protein analysis. The results showed modulation of Wnt signaling, following CXL treatment, as measured by the protein levels of Wnt7b and Wnt10a, correlated to the expression of α-smooth muscle actin (SMA). Further, the expression of a recently identified KC biomarker candidate, prolactin-induced protein (PIP), was positively impacted by CXL in HKCs. CXL-driven upregulation of PGC-1 and the downregulation of SRC and Cyclin D1 in HKCs were also noted. Although the cellular/molecular impacts of CXL are largely understudied, our studies provide an approximation to the complex mechanisms of KC and CXL. Further studies are warranted to determine factors influencing CXL outcomes.

## 1. Introduction

Keratoconus (KC) is a common ectatic corneal dystrophy that causes thinning, bulging, and scarring of the human cornea [1]. While the disease progression and severity can vary significantly among individuals, it is estimated that approximately 1:400 people are affected worldwide [2]. The first KC treatments described relied on concave lenses or the equivalent hard contact lens used today in order to correct refractive errors. It was posited in these early studies that structural defects in the central cornea led to the formation of a cone centrally due to normal intraocular pressure. Though the cause of KC is still unknown today, these early clinical studies describe similar medical approaches compared to modern times in the treatment of KC.

Early-stage KC is typically managed with spectacles and contact lenses [3]. The best-corrected visual acuity (BCVA) in mild–moderate stages may be managed with intrastromal corneal ring segments (ICRS) [4] to achieve corneal flattening; however, this does not stop disease progression for most patients. Recently, there has been a wave of interest in other corneal inlay procedures, such as Bowman Layer transplantation (BL) and corneal allogenic intrastromal ring segments (CAIRS) [5]. In late-stage KC, corneal transplantation (deep anterior lamellar keratoplasty (DALK) or penetrating keratoplasty (PKP)) [6] becomes the only option for visual rehabilitation. Given the short-term and long-term risks associated with corneal transplants, there is a significant need for treatment options in mild–moderate disease stages to halt disease progression and avoid corneal surgery.

Since 2007, collagen crosslinking (CXL) has been used internationally to halt KC progression in mild–moderate cases [7]. In the United States, the epithelium-off technique (Dresden protocol) has been utilized by clinicians since FDA approval in 2016 [8]. CXL is a non-invasive procedure using riboflavin (vitamin B2) eye drops and ultraviolet type A (UV-A) light to promote corneal tissue stiffness and stabilize ectatic disease. While CXL can halt KC disease progression and obviate the need for corneal transplantation, not all patients with KC may qualify for CXL under current protocols. Currently, KC patients must demonstrate “progression” of disease using objective criteria, such as an increase in steep keratometry, an increase in the refractive cylinder, or loss of best-corrected visual acuity (BCVA) [9]. As such, a significant number of patients with mild disease who would benefit from CXL may not meet the established criteria for CXL until, ironically, the disease worsens. Additional criteria must also be satisfied before qualifying for CXL: patients older than 16 years, with a minimum corneal thickness (CCT) of 400 microns, maximal keratometry < 60 D, and no other known corneal disease [10]. Finally, there are a growing number of patients with post-LASIK ectasia [11] who may also benefit from CXL to arrest disease progression but may not qualify due to the criteria mentioned above. At present, there is significant room for improvement in techniques and expanding the range of eligible patients to increase CXL safety and stabilization effects [12,13].

It is well known that extracellular matrix (ECM) stiffness regulates cellular phenotypes and behaviors, including proliferation, differentiation, apoptosis, alignment, and migration [14]. Surprisingly, the mechanism underlying the sensing of CXL-driven mechanical cues by the corneal stromal cells and subsequent elasticity-triggered pathways remains largely unknown. Our group previously introduced a 3D self-assembled in vitro CXL model as a novel tool for investigating and unraveling the effects of CXL at the cellular and molecular levels [15,16,17]. Those findings revealed key differences in ECM organization and degradation, proteoglycan expressions, fibrosis expression, SMAD downstream signaling pathway expressions, and metabolism, following 4 weeks of CXL treatment. Our current study investigated how differences in ECM thickness (2 versus 4 weeks) modulate Wnt signaling, fibrosis, PIP, and intermediate targets (PGC1, Src, and Cyclin D), following CXL.

The Wnt signaling pathway plays a major role in normal corneal development, with Wnt7b [18] and Wnt10a [19] reported to be associated with an increased risk of KC development [20,21]. Furthermore, thinner KC corneas have been linked to a higher risk of fibrotic development following CXL treatment [22]. PIP has been reported to be involved with corneal structural stability [23] and as a biomarker for KC [24]. Previous studies have also implicated PGC-1 [25], Src [26], and cyclin D [27] as playing important roles in the corneal ECM, proliferation, and repair process. Given that there is no KC animal model and 2D cell culture setups cannot be used for CXL investigations (i.e., no ECMs), we sought to investigate the effect of CXL on 3D self-assembled ECMs using primary human keratoconus stromal cells (HKCs) compared to their healthy counterparts (HCFs). In this study, we “mirror” the concept of ECM thinning by applying CXL at different time points of the assembled ECM (weeks 2 and 4) and determining the modulation of specific downstream targets.

There has been a notable scarcity of laboratory studies on the ultrastructural alterations and microenvironment of KC-treated corneas. The vast majority of studies are focusing on the biomechanical effects of CXL, lacking critical information on items such as corneal cellular responses, extracellular matrix (ECM) degradation, and reactive oxygen species generation that could collectively provide valuable information.

How CXL affects the cellular machinery of the human cornea (both in the short and long term) is under-investigated due to the lack of sufficient models. The data shown in this study provide a better understanding of CXL. In the future, such information may assist in addressing the clinical need for a new, modified CXL protocol(s) for previously disqualified corneas, particularly with CCT < 400 microns. Our previous published work highlights the importance and novelty of our in vitro approaches [15,16,17], whereas the data reported herein indicates its potential.

## 2. Materials and Methods

### 2.1. Ethics and Donor Information

This study adhered to the tenets of the Declaration of Helsinki. Healthy human corneas were obtained from the National Disease Research Interchange (NDRI, Philadelphia, PA). KC donor corneas were obtained from our clinical collaborator (KMR; Dean McGee Eye Institute, Oklahoma City, OK, USA) with Institutional Research Board (IRB) approval (IRB# 10108). Written informed consents were obtained from all participants. Healthy corneas, obtained from the National Disease Research Interchange (NDRI), adhered to inclusion criteria: absence of KC diagnosis with no ocular, corneal, or systemic diseases. Inclusion criteria for KC patients required a documented KC diagnosis by a certified ophthalmologist and the absence of other ophthalmic conditions. Patients with a history of CXL, DALK, or PKP were excluded from this study.

### 2.2. Cell Isolation and 3D Self-Assembled Constructs

Primary corneal fibroblasts were isolated from healthy and KC human corneas, as previously described [28,29]. Briefly, after brief scraping with a razor blade, the endothelium and epithelium were removed from the sample corneal specimen. The remaining stromal tissue was cut into small pieces (approximately 2 mm × 2 mm) and allowed to adhere to the bottom of a T25 flask for 30 min at 37 °C before slowly adding Eagle’s Minimum Essential Media (EMEM: ATCC: Manassas, VA, USA), containing 10% fetal bovine serum (FBS: Atlantic Biologic’s; Lawrenceville, CA, USA) and 1% antibiotic/antimycotic solution (Gibco^®^ Antibiotic-Antimycotic, Life technologies, Grand Island, NY, USA), without disturbing the explants. At 80–90% confluency, explants were further passaged and expanded. All experiments were executed with cells cultured for no longer than passage 7.

To create the 3D constructs, we utilized our previously described self-assembled ECM model [30,31]. Briefly, HCFs and HKCs were seeded on transwell 6-well plates with polycarbonate membrane inserts with 0.4-μm pores (Transwell; Corning Costar; Charlotte, NC, USA) at a density of 1 × 10^6^ cells/well and cultured in 1.5 mL of 10% FBS EMEM medium and 1% antibiotic stimulated with 0.5 mM 2-O-α-D-glucopyranosyl-L-ascorbic acid (Vitamin C, American Custom Chemicals Corporation, San Diego, CA, USA) in both top and bottom wells. Fresh media was supplied every other day. Two experimental groups were utilized in an attempt to “mirror” thinner (=2-week cultures) and thicker (=4-week cultures) ECMs. All experimental conditions were repeated at least 3 times (n = 3).

### 2.3. Collagen Crosslinking (CXL)—In Vitro

Utilizing our previously established 3D CXL model [15,16,17], a 0.1% solution of riboflavin and PBS was added to the 3D constructs, followed by UVA irradiation after 2 and 4 weeks. UVA irradiation was delivered with a UV-X illumination system (v.1000; IROC AG, Zurich, Switzerland) at a wavelength of 360–370 nm and an irradiance of 3 mW/cm^2^ of UVA, with a total energy dose of 5.4 J/cm^2^. UV-X illumination system calibration was performed before each treatment using a UVA meter (LaserMate-Q; LASER 2000, Wessling, Germany). For each well/construct, UVA irradiation was administered for 3 min and at a 3 cm distance, as previously optimized for our in vitro studies. Following irradiation, each construct was rinsed 3X with PBS and incubated with fresh media for 12h to promote post-radiation repair for both 2- and 4-week groups.

### 2.4. Protein Isolation and Western Blots

Protein was extracted and isolated from all constructs with their purities determined utilizing a Pierce™ BCA Protein Assay kit (ThermoFisher Scientific; Rockford, IL, USA). Western blot analyses were performed by loading 20 ug of protein per well in Precast Novex 4–20% Tris-Glycine Mini Gels (Life Technologies; Carlsbad, CA, USA). Electrophoresis was performed on the protein-loaded gels and then transferred onto nitrocellulose membranes (Bio-Rad Laboratories, Inc; Hercules, CA, USA). Following one-hour incubation in 5% BSA blocking solution at room temperature (ThermoFisher Scientific; Rockford, IL, USA), the protein-loaded membranes were incubated with primary antibodies overnight at 4 °C with the following antibodies: anti-alpha smooth muscle actin (ab5694), anti-Src (ab47405), anti-Wnt7b (ab94915), anti-Wnt10a (ab106522), anti-cyclin D1 (ab226977), anti-PGC1 (ab54481), and anti-PIP (ab133290). Subsequently, the membranes were washed in TBST and then incubated in a secondary antibody, Alexa Flour^®^ 568 Donkey anti-Rabbit, IgG (H^+^L) (Life Technologies; Carlsbad, CA, USA) for one hour at room temperature. Lastly, the membranes were imaged using the UVP imaging system (Analytik Jena US LLC; Upland, CA, USA) with signal detection acquired via VisionWorks™LS Image Acquisition and Analysis Software (Analytik Jena US LLC; Upland, CA, USA). The results for each target were analyzed by adjusting the total density values of individual bands to those of the housekeeping antibody, anti-GAPDH (Supplemental Figure S1; ab9485; Abcam; Cambridge, MA, USA). Further, each condition was then normalized to HCF controls, and expressions for each antibody were plotted and analyzed for statistical analysis.

### 2.5. Statistical Analysis

Data (mean ± SEM) statistical significance were analyzed via one-way ANOVA (GraphPad Prism 9 software; La Jolla, CA, USA). Differences in expression were considered statistically significant when *p* < 0.05. * *p* < 0.05, ** *p* < 0.01, *** *p* < 0.001, **** *p* < 0.0001.

## 3. Results

### 3.1. Signaling Pathways

**Figure 1:**
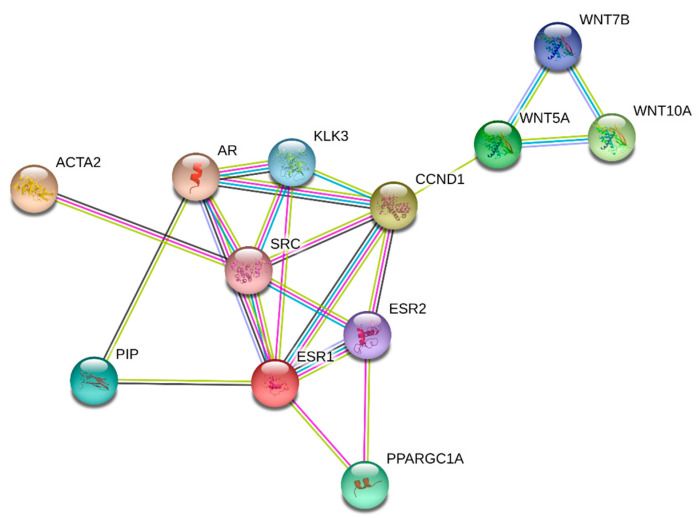
STRING analysis displaying WNT5A, WNT7B, WNT10A, CCND1, ESR1, ESR2, PPARGC1A, SRC, PIP, KLK3, AR, and ACTA2 protein associations.

In this study, the objective was to determine the key interactions and pathways altered in HKCs following CXL in the context of our previous discoveries. Given that the signaling mechanisms of CXL are largely unknown, we performed a STRING analysis. STRING is an established database of known and predicted protein–protein interactions, including direct (physical) and indirect (functional) associations. Figure 1 shows that Wnt5a, Wnt7b, and Wnt10a are linked to estrogen/androgen receptors (ESR1/2 and AR), prolactin-induced protein (PIP), and smooth muscle actin (ACTA2) via a path that includes cyclinD1, non-receptor tyrosine kinase SRC, peroxisome proliferator-activated receptor gamma coactivator 1 (PGC-1), and kallikrein-3 (KLK3). This is very intriguing to us given our previous in vitro and in vivo studies on sex hormones, PIP, ACTA2, and their roles in KC. Based on the STRING analysis, we utilized our in vitro CXL model and investigated the expression of specific targets.

### 3.2. Wnt Signaling and Fibrosis

Wnt signaling has only recently been linked to KC [32,33], while corneal fibrosis and its markers are known factors in the pathobiology of this disease. Thus, we investigated the modulation of Wnt7b (also see Supplemental Figure S2), Wnt10a (also see Supplemental Figure S3), and SMA (also see Supplemental Figure S4) using our CXL in vitro model.

**Figure 2:**
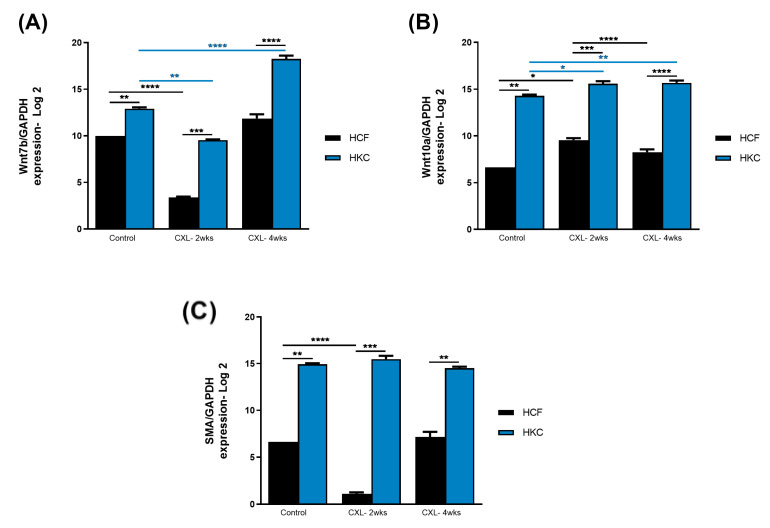
Protein expression of (**A**) Wnt7b, (**B**) Wnt10a, and (**C**) SMA in HCF and HKC 3D constructs following CXL treatment after 2 and 4 weeks. Constructs without treatment serve as controls. * represents *p* < 0.05, ** represents *p* < 0.01, *** represents *p* < 0.001, and **** represents *p* < 0.0001.

Figure 2A shows the protein expression of Wnt7b in HCFs and HKCs pre- and post-CXL. Wnt7b expression was significantly higher in HKC controls when compared to HCF controls. Interestingly, both the HCF- and HKC-2wks CXL constructs showed significant downregulation of Wnt7b when compared to their respective controls. The HKC-4wks CXL constructs showed a significant Wnt7b upregulation (Figure 2A), indicating ECM thickness is critical to its modulation. Wnt10a expression (Figure 2B) was significantly higher in HKC controls when compared to HCF controls, which was similar to Wnt7b. Moderate but significant upregulation of Wnt10a was observed in HKC-2wks and -4wks CXL compared to HKC controls. Conversely, HCF-4wks CXL demonstrated downregulation of Wnt10a compared to HCF-2wks CXL (Figure 2B). Lastly, SMA expression (a fibrotic marker) was significantly downregulated in HCF-2wks CXL constructs (Figure 2C) but did not change in HKCs, which showed higher levels of SMA at all conditions when compared to HCFs. These data suggest the Wnt signaling pathway is mostly activated (or upregulated) upon CXL regardless of the ECM thickness with the exception of Wnt7b, which seems to be not only CXL but also ECM-dependent. Furthermore, the modulation of Wnt signaling appears to correlate with SMA levels.

### 3.3. Prolactin-Induced Protein

We previously reported that PIP is a biomarker for KC, as measured by its levels in biological fluids (saliva, blood, and tears) [34]. In those clinical studies, the expression of PIP was significantly downregulated in KC patients, regardless of the fluid tested. Our STRING analysis (Figure 1) shows a direct link between PIP and Wnt pathway targets. Thus, we investigated PIP (also see Supplemental Figure S5) expression pre- and post-CXL.

**Figure 3:**
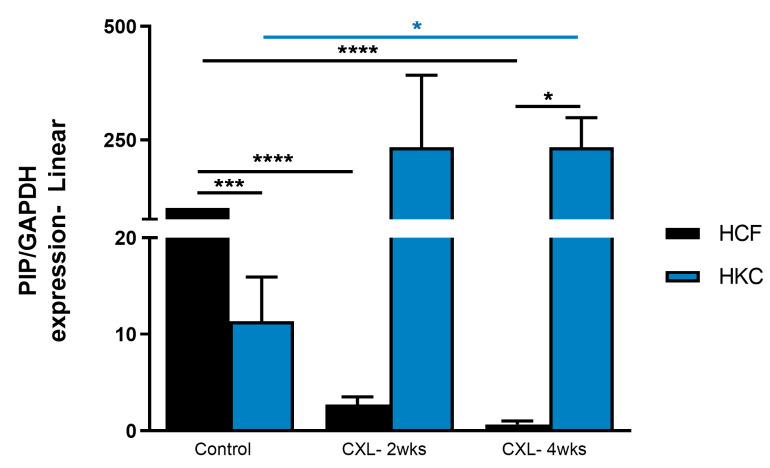
Protein expression of PIP in HCF and HKC 3D constructs following CXL treatment after 2 and 4 weeks. Constructs without treatment serve as controls. * represents *p* < 0.05, *** represents *p* < 0.001, and **** represents *p* < 0.0001.

Figure 3 shows significant downregulation of protein expression of PIP in HCFs and HKCs pre- and post-CXL. This trend was observed in both HCF-2wks and HCF-4wks CXL constructs when compared to their corresponding controls. In contrast, CXL led to the recovery of PIP expression to “healthy” levels in HKC-2wks and HKC-4wks CXL constructs, suggesting a possible “recovery” mechanism. Thus, PIP appears to play a critical role in the KC cornea microenvironment and is modulated by CXL.

### 3.4. Intermediate Targets

**Figure 4:**
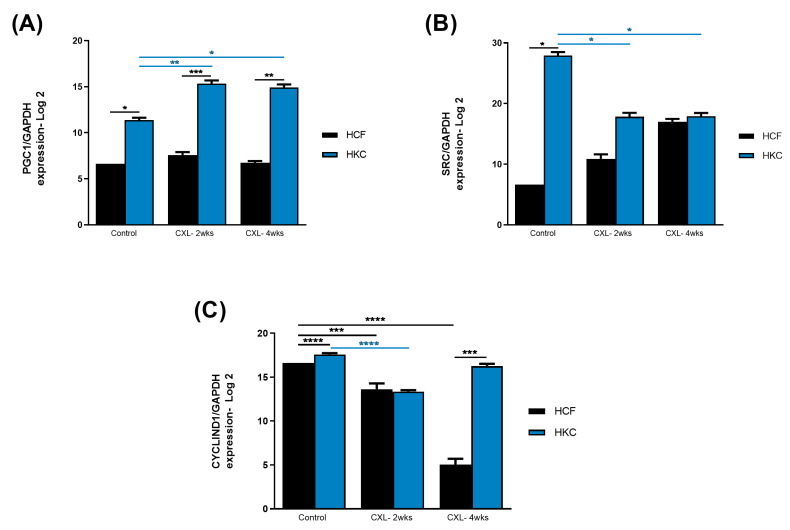
Protein expression of (**A**) PGC1, (**B**) SRC, and (**C**) cyclin D1 in HCF and HKC 3D constructs following CXL treatment after 2 and 4 weeks. Constructs without treatment serve as controls. * represents *p* < 0.05, ** represents *p* < 0.01, *** represents *p* < 0.001, and **** represents *p* < 0.0001.

Based on the STRING analysis (Figure 1), we also investigated the expression of PGC-1 (also see Supplemental Figure S6), SRC (also see Supplemental Figure S7), and cyclin D1 (also see Supplemental Figure S8). Figure 4A shows the protein expression of PGC-1 (peroxisome proliferator-activated receptor gamma coactivator 1, a transcriptional coactivator that regulates the genes involved in energy metabolism). PGC-1 expression was not affected in HCFs following CXL. However, HKCs showed significant upregulation of PGC-1 expression both at 2- and 4-week CXL compared to HKC controls, as well as in all HCF conditions. Figure 4B shows the protein expression of SRC (proto-oncogene, non-receptor tyrosine kinase; also called c-Src). SRC expression was significantly higher in HKC controls when compared to HCF controls. However, upon CXL application, SRC expression was significantly downregulated in both HKC-2wks and HKC-4wks constructs. In HCFs, SRC expression, upon CXL, showed upregulation but did not reach a significant threshold. Cyclin D1 (a member of the highly conserved cyclin family; activity is required for cell cycle G1/S transition and cell cycle progression) protein expression is shown in Figure 4C. Cyclin D1 expression was significantly downregulated at 2-week CXL for both HCFs and HKCs. However, at 4-week CXL, cyclin D1 expression recovered in HKCs but was further downregulated in HCFs indicating opposing mechanisms between the two cell types.

## 4. Discussion

Corneal transplantation has an extremely high short-term success rate of 90% first-year graft survival, with long-term graft survival reducing to 62% at 10 years post-surgery and down to 35% in patients with active ocular inflammation. Thus, many young KC patients may require multiple corneal transplants during their lifetime.

While CXL is an effective treatment modality for progressive KC, there are numerous areas remaining for the improvement and expansion of this procedure for patients beyond the approved protocols. For example, in the United States, conventional CXL is approved only in eyes with a corneal thickness of at least 400 microns [10], which excludes a significant number of KC patients who may potentially benefit from this therapy. Further, painful epithelial defects with secondary corneal infection and scarring resulting from epithelial removal prior to CXL treatment have also been reported. Therefore, techniques to expand the inclusion criteria for safe and effective CXL are an area of interest for researchers and clinicians alike.

The long-term outcomes of CXL are still under investigation given the relatively short time since the modality was approved and widely adopted. Despite very promising results, treatment failures and progression of corneal ectasia have been reported after CXL. Though CXL is far from the sine qua non-KC treatment, it remains an effective option for clinicians as few alternative therapies exist. Thus, the optimization of CXL, including improving efficacy and expanding inclusion criteria, is an area of significant interest. CXL optimization and adaptation to patients’ needs are lacking for several reasons: (1) the lack of a reliable KC animal model that will allow in vivo, non-human examination of altered CXL modes and (2) the lack of understanding of the molecular cascades triggered by CXL. In our study, we used our established 3D in-vitro model to simulate thin (week 2 thinner ECM) and thick corneas (week 4 thicker ECM) to determine the effects of CXL in both types of tissue. Given the numerous “unknowns” in CXL molecular machinery, the present study was guided by and executed based on known interactions determined by STRING analysis (Figure 1). We focused our studies on PIP, Wnt7b, Wnt10, SMA, PGC1, SRC, and CYCLID1, representing a slew of molecular signaling pathways. However, further studies are warranted to dive deeper into these complex machineries and fully delineate their role in CXL.

There are likely many, but currently unknown, clinical implications of characterizing how CXL changes corneal tissue at the molecular and cellular levels. Wnt signaling modulation has been reported in KCs [19,33,35,36], largely focused on the epithelium and through RNA-Seq approaches. Interestingly, Cuellar-Partida linked an exonic Wnt10A variant to the central corneal thickness. To date, the role and modulation of the Wnt pathway in CXL are not reported. Based on our findings, Wnt-related targets are upregulated following CXL, possibly providing insight into the corneal response to treatment. In the literature, single nucleotide polymorphisms (SNPs) of Wnt7b were reported to be associated with central corneal thickness (CCT) [18]. Additionally, Wnt7b has also been linked to inflammation and fibrosis; for example, in the wounded cornea, Wnt7b is upregulated in the central cornea and is known to promote corneal epithelial cell proliferation and wound closure mainly via the β-catenin pathway [37]. Kulkarni et al. [38] showed the downregulation of Wnt7b in corneal epithelial cells with overexpressed miR-10b and up-regulation upon miR-10b inhibition. Their study suggested that miR-10b plays a significant role in limbal epithelial stem cell proliferation and their differentiation to transient amplifying cells. More recently, our group showed local inhibition of Wnt signaling in the cornea by kallistatin, an LRP6-blocking antibody or the soluble VLDL receptor ectodomain (an endogenous Wnt signaling inhibitor) delayed wound healing [39]. In recent years, there has been a growing understanding of how Wnt is implicated in corneal health and disease. Taken together, our study highlights the involvement of Wnt signaling with CXL and possibly indicates the existence of an intricate mechanism that needs to be further studied.

By strengthening the ECM, CXL arrests KC progression. However, the exact mechanisms by which these effects are sensed by the resident cells and the resulting various cellular responses elicited still need to be fully understood. The contributions of the ECM to the physiologic actions of prolactin are increasingly investigated; however, very little is known about the functional relationship between ECM and prolactin signaling in CXL. We have shown the modulation of PIP in health and disease in vivo (human biological fluids) [34,40,41]. We have also shown the function of PIP in the corneal microenvironment using a mouse ocular burr wound model, where PIP showed noteworthy anti-fibrotic potentiality. In this study, we investigated the role of PIP in the context of CXL. PIP and CXL appear to be cell-type dependent as PIP expression was downregulated in HCFs but upregulated in HKCs. The “rescuing” of PIP levels could be significant concerning CXL mechanistic actions. SRC is known to influence adhesion stabilization on carcinoma cells to ECM [42] and is also reported to impact corneal wound healing. Interestingly, SRC KO mice exhibit bilateral central cornea defects [43]. In the context of KC, SRC has received limited attention. You et al. [35] reported SRC’s significant upregulation in KC corneal epithelium compared to the control group. Our studies demonstrate significant upregulation of SRC in HKCs compared to HCFs, followed by significant downregulation due to CXL treatment.

Modulation of PGC-1 by CXL is also notable as key cellular signals that control energy and nutrient homeostasis (i.e., cAMP, cytokines, etc.) strongly activate PGC-1, leading to the stimulation of mitochondrial oxidation. Further, PGC-1 is a master regulator of ROS scavenging enzymes [44,45], which are known to be present in the KC cornea. Such cellular activities are almost central to the apoptosis and mitochondrial dysfunction noted in KC pathobiology, and thus CXL protocols might have to be adjusted accordingly.

Like any other model, limitations exist, such as a lack of endothelial layer and no mechanical action removal of the epithelium prior to CXL. Epithelial detachment/removal during CXL is a critical event and is currently mirrored in our model. Thus, further studies are warranted in order to understand the mechanisms involved, including epithelium proliferation and the role of limbal stem cells during healing post-CXL. Nonetheless, the findings from the current study are critical, given the lack of any animal model(s) and/or an alternative in vitro model. Future studies are warranted to analyze longer phase time points and further compare different CXL treatment strategies to best determine how signaling pathways are modulated. While in vivo studies are of interest, we currently do not have a KC model, which limits such studies to healthy corneas only and does not accurately represent the cellular and molecular impact of CXL. Insights into the molecular pathways of KC and CXL may ultimately expand treatment options for patients suffering from KC. Furthermore, studies should be expanded to larger cohort(s) in order to increase the significance of the above-mentioned mechanisms.

Overall, our study is the first attempt to mirror “thinner” corneal ECMs (in vitro) and determine the impact of CXL. Unfortunately, cellular/molecular studies related to CXL are lacking. Thus, the investigation of such mechanisms would provide an opportunity to better understand how CXL works at the cellular level and perhaps improve the modality in the future.

## Data Availability

The data presented in this study are available on request from the corresponding author.

## Supplementary Materials

The following supporting information can be downloaded at: https://www.mdpi.com/article/10.3390/biom13040696/s1, Figure S1: Western Blot images and Total Density values of GAPDH protein expression in HCF and HKC 3D constructs following CXL treatment after 2 and 4 weeks. Constructs without treatment serve as controls. Each condition was repeated 4 times; Figure S2: Western Blot images and Total Density values of Wnt7b protein expression in HCF and HKC 3D constructs following CXL treatment after 2 and 4 weeks. Constructs without treatment serve as controls. Each condition was repeated 4 times; Figure S3: Western Blot images and Total Density values of Wnt10a protein expression in HCF and HKC 3D constructs following CXL treatment after 2 and 4 weeks. Constructs without treatment serve as controls. Each condition was repeated 4 times; Western Blot images and Total Density values of SMA protein expression in HCF and HKC 3D constructs following CXL treatment after 2 and 4 weeks. Constructs without treatment serve as controls. Each condition was repeated 4 times; Figure S4: Western Blot images and Total Density values of SMA protein expression in HCF and HKC 3D constructs following CXL treatment after 2 and 4 weeks. Constructs without treatment serve as controls. Each condition was repeated 4 times; Figure S5: Western Blot images and Total Density values of PIP protein expression in HCF and HKC 3D constructs following CXL treatment after 2 and 4 weeks. Constructs without treatment serve as controls. Each condition was repeated 4 times; Figure S6: Western Blot images and Total Density values of PGC1 protein expression in HCF and HKC 3D constructs following CXL treatment after 2 and 4 weeks. Constructs without treatment serve as controls. Each condition was repeated 4 times; Figure S7: Western Blot images and Total Density values of SRC protein expression in HCF and HKC 3D constructs following CXL treatment after 2 and 4 weeks. Constructs without treatment serve as controls. Each condition was repeated 4 times; Figure S8: Western Blot images and Total Density values of CyclinD1 protein expression in HCF and HKC 3D constructs following CXL treatment after 2 and 4 weeks. Constructs without treatment serve as controls. Each condition was repeated 4 times.
