# Supplementary material for: Collagen Crosslinking for Keratoconus: Cellular Signaling Mechanisms
Source: Biomolecules. 2023 Apr 20;13(4):696. doi: 10.3390/biom13040696 (PMC10135890; doi:10.3390/biom13040696)
Supplement: Supplementary file 1 [file biomolecules-13-00696-s001.zip › biomolecules-2244992-supplementary.pdf]

GAPDH molecular weight- 37 kDa; **MagicMark ladder molecular weights** = 20, 30, 40, 50, 60, 80, 100, 120, and 220 kDa

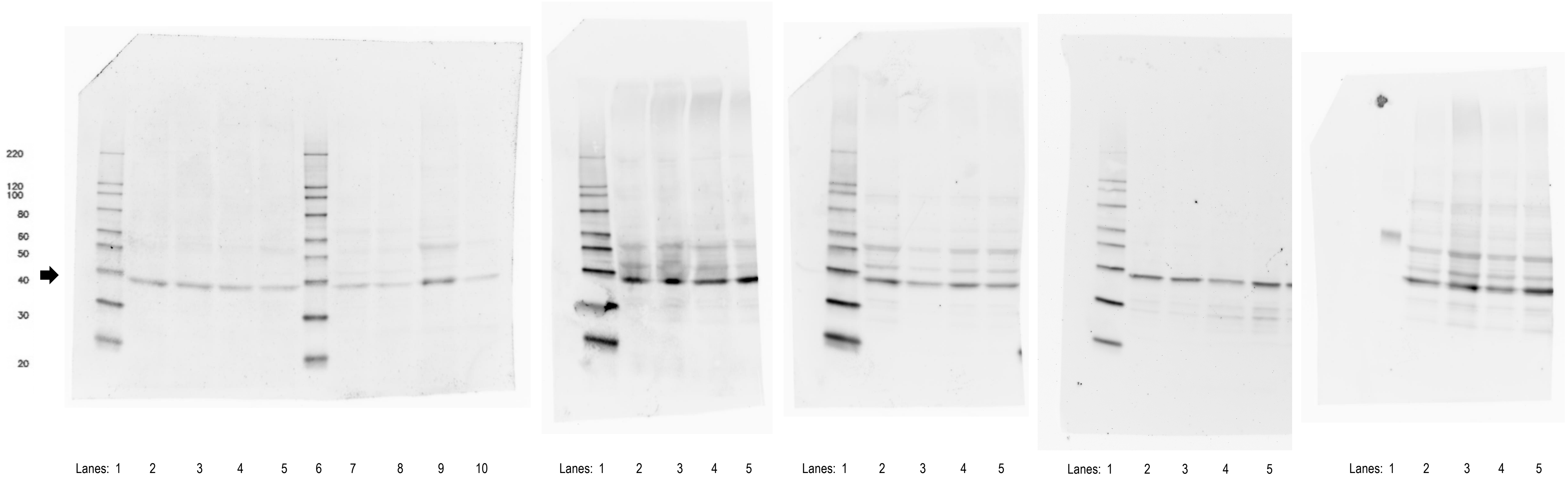

*Ladder (lanes 1 & 6);
HCF control (lanes 2-5)
HKC control (lanes 7-10)

*Ladder (lane 1);
HCF+CXL week 2 (lanes 2-5)

*Ladder (lane 1);
HKC+CXL week 2 (lanes 2-5)

*Ladder (lane 1);
HCF+CXL week 4 (lanes 2-5)

*Ladder (lane 1);
HKC+CXL week 4 (lanes 2-5)

GAPDH	Total Density					
	HCF control	HKC control	HCF+CXL 2 week	HKC+CXL 2 week	HCF+CXL 4 week	HKC+CXL 4 week
sample 1	7.33E+07	1.33E+05	7.65E+07	9.64E+05	7.94E+07	2.01E+05
sample 2	6.23E+07	1.23E+05	6.57E+07	6.57E+05	7.82E+07	2.78E+05
sample 3	6.17E+07	6.17E+05	6.02E+07	1.02E+05	8.52E+07	1.23E+05
sample 4	7.12E+07	9.12E+05	6.05E+07	1.05E+05	8.62E+07	3.30E+05

Supplemental Figure S1. Western Blot images and Total Density values of GAPDH protein expression in HCF and HKC 3D constructs following CXL treatment after 2 and 4 weeks. Constructs without treatment serve as controls. Each condition was repeated 4 times.

Wnt7b molecular weight- 39 kDa; **MagicMark ladder molecular weights** = 20, 30, 40, 50, 60, 80, 100, 120, and 220 kDa

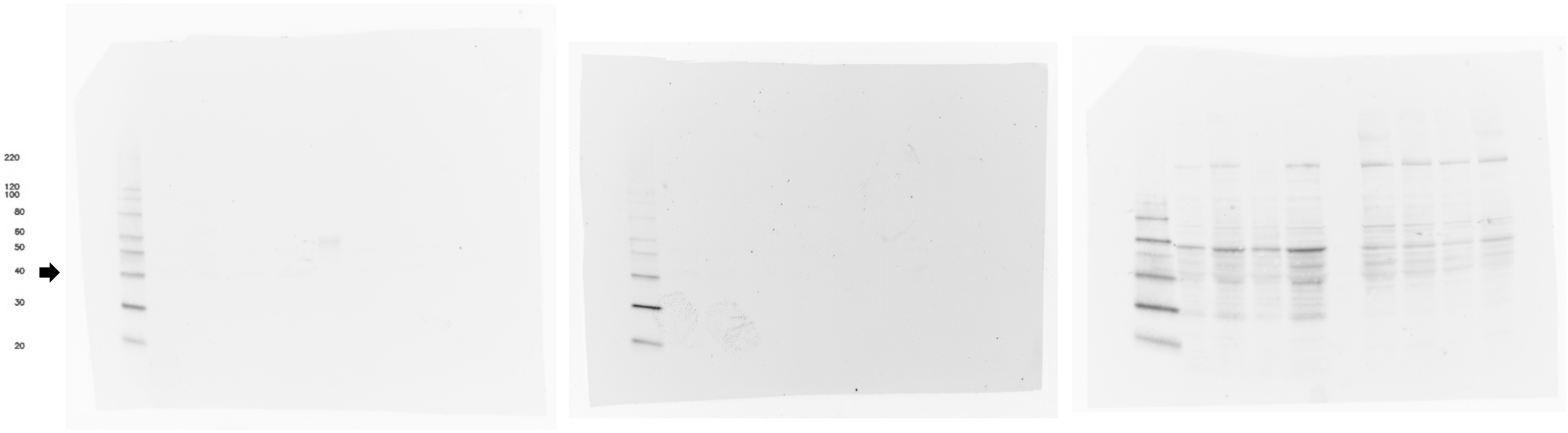

Lanes: 1 2 3 4 5 6 7 8 9 10

*Ladder (lane 1 & 6);
HCF control (lanes 2-5)
HKC control (lanes 7-10)

Lanes: 1 2 3 4 5 6 7 8 9 10

*Ladder/Blank (lane 1 & 6);
HCF+CXL week 2 (lanes 2-5)
HKC+CXL week 2 (lanes 7-10)

Lanes: 1 2 3 4 5 6 7 8 9 10

*Ladder/Blank (lane 1 & 6);
HCF+CXL week 4 (lanes 2-5)
HKC+CXL week 4 (lanes 7-10)

Wnt7b	Total Density					
	HCF control	HKC control	HCF+CXL 2 week	HKC+CXL 2 week	HCF+CXL 4 week	HKC+CXL 4 week
sample 1	2.49E+06	3.49E+05	5.43E+04	2.43E+04	1.92E+06	8.92E+06
sample 2	5.38E+06	2.88E+05	6.10E+04	3.10E+04	2.00E+07	8.00E+06
sample 3	5.35E+06	2.35E+05	5.67E+04	2.67E+04	4.57E+07	3.17E+06
sample 4	8.03E+06	2.13E+05	4.58E+04	2.58E+04	2.83E+07	3.03E+06

Supplemental Figure S2. Western Blot images and Total Density values of Wnt7b protein expression in HCF and HKC 3D constructs following CXL treatment after 2 and 4 weeks. Constructs without treatment serve as controls. Each condition was repeated 4 times.

Wnt10a molecular weight- 46 kDa; **MagicMark ladder molecular weights** = 20, 30, 40, 50, 60, 80, 100, 120, and 220 kDa

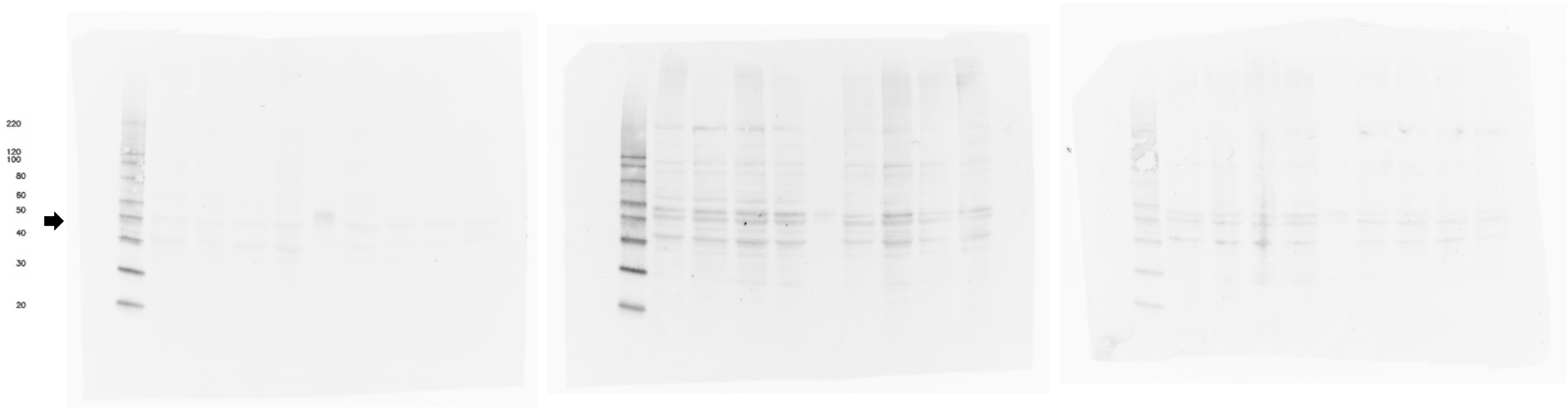

Lanes: 1 2 3 4 5 6 7 8 9 10

*Ladder (lane 1 & 6);
HCF control (lanes 2-5)
HKC control (lanes 7-10)

Lanes: 1 2 3 4 5 6 7 8 9 10

*Ladder (lane 1 & 6);
HCF+CXL week 2 (lanes 2-5)
HKC+CXL week 2 (lanes 7-10)

Lanes: 1 2 3 4 5 6 7 8 9 10

*Ladder (lane 1 & 6);
HCF+CXL week 4 (lanes 2-5)
HKC+CXL week 4 (lanes 7-10)

Wnt10a	Total Density					
	HCF control	HKC control	HCF+CXL 2 week	HKC+CXL 2 week	HCF+CXL 4 week	HKC+CXL 4 week
sample 1	1.49E+06	1.49E+06	9.43E+06	6.43E+06	3.92E+06	1.92E+06
sample 2	1.38E+06	2.38E+06	1.10E+07	3.10E+06	6.00E+06	2.00E+06
sample 3	2.35E+06	2.35E+06	1.67E+07	3.67E+06	9.57E+06	3.57E+06
sample 4	1.03E+06	2.03E+06	8.58E+06	7.58E+06	3.83E+06	3.83E+06

Supplemental Figure S3. Western Blot images and Total Density values of Wnt10a protein expression in HCF and HKC 3D constructs following CXL treatment after 2 and 4 weeks. Constructs without treatment serve as controls. Each condition was repeated 4 times.

SMA molecular weight- 42 kDa; **MagicMark ladder molecular weights** = 20, 30, 40, 50, 60, 80, 100, 120, and 220 kDa

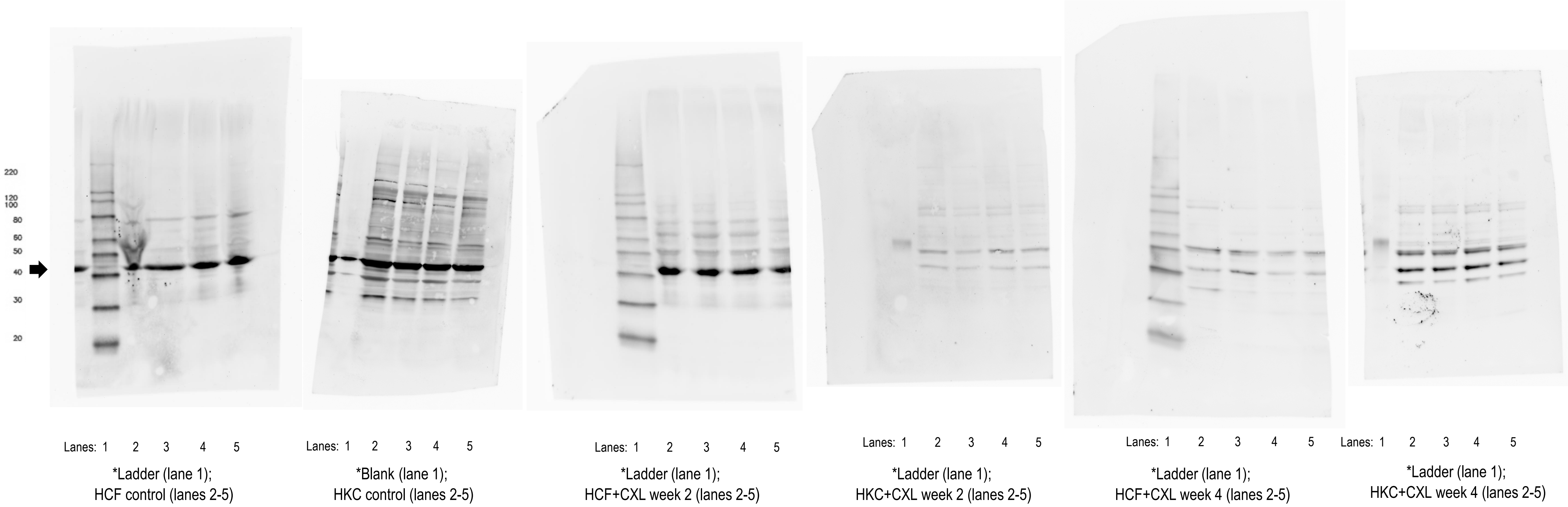

SMA	Total Density					
	HCF control	HKC control	HCF+CXL 2 week	HKC+CXL 2 week	HCF+CXL 4 week	HKC+CXL 4 week
sample 1	4.49E+07	3.69E+07	4.63E+05	1.13E+07	1.92E+07	1.72E+07
sample 2	1.13E+07	4.08E+07	4.60E+05	6.40E+07	2.60E+07	1.60E+07
sample 3	1.05E+07	3.35E+07	4.27E+05	8.37E+07	7.87E+07	1.87E+07
sample 4	1.03E+07	4.83E+07	2.58E+05	7.98E+07	1.13E+07	1.03E+07

Supplemental Figure S4. Western Blot images and Total Density values of SMA protein expression in HCF and HKC 3D constructs following CXL treatment after 2 and 4 weeks. Constructs without treatment serve as controls. Each condition was repeated 4 times.

PIP molecular weight- 17 kDa; **MagicMark ladder molecular weights** = 20, 30, 40, 50, 60, 80, 100, 120, and 220 kDa

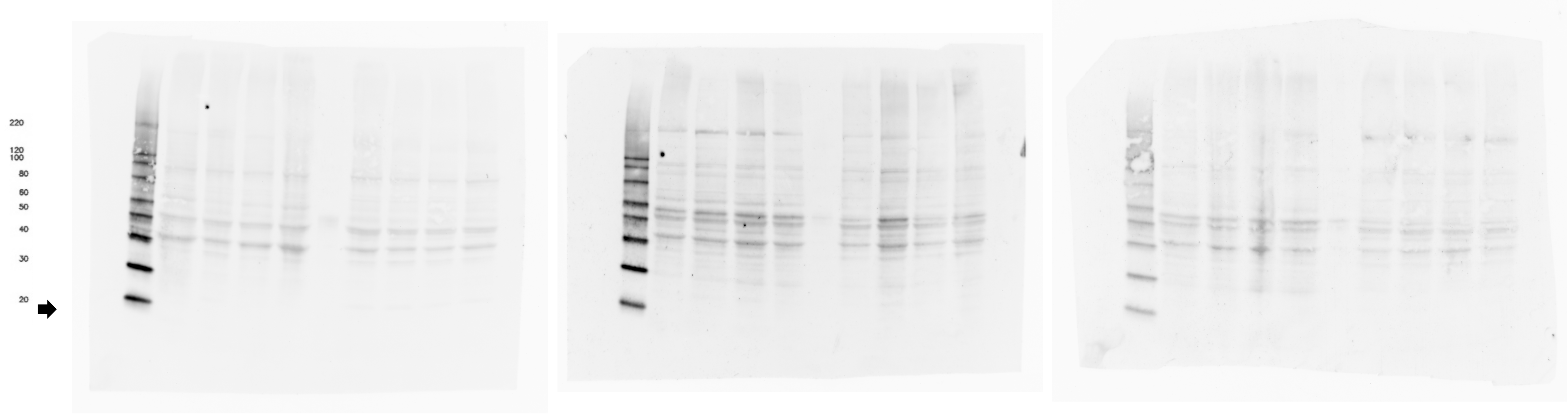

Lanes: 1 2 3 4 5 6 7 8 9 10

*Ladder (lane 1 & 6);
HCF control (lanes 2-5)
HKC control (lanes 7-10)

Lanes: 1 2 3 4 5 6 7 8 9 10

*Ladder (lane 1 & 6);
HCF+CXL week 2 (lanes 2-5)
HKC+CXL week 2 (lanes 7-10)

Lanes: 1 2 3 4 5 6 7 8 9 10

*Ladder (lane 1 & 6);
HCF+CXL week 4 (lanes 2-5)
HKC+CXL week 4 (lanes 7-10)

PIP	Total Density					
	HCF control	HKC control	HCF+CXL 2 week	HKC+CXL 2 week	HCF+CXL 4 week	HKC+CXL 4 week
sample 1	2.73E+07	1.22E+04	3.90E+05	3.26E+05	3.01E+05	6.41E+05
sample 2	8.31E+07	1.01E+04	1.20E+06	2.21E+06	1.09E+05	4.23E+05
sample 3	2.71E+07	4.99E+04	2.13E+06	2.67E+05	9.58E+05	2.48E+05
sample 4	3.21E+07	6.63E+04	1.17E+06	1.12E+05	1.27E+05	1.74E+05

Supplemental Figure S5. Western Blot images and Total Density values of PIP protein expression in HCF and HKC 3D constructs following CXL treatment after 2 and 4 weeks. Constructs without treatment serve as controls. Each condition was repeated 4 times.

PGC1 molecular weight- 92 kDa; **MagicMark ladder molecular weights =** 20, 30, 40, 50, 60, 80, 100, 120, and 220 kDa

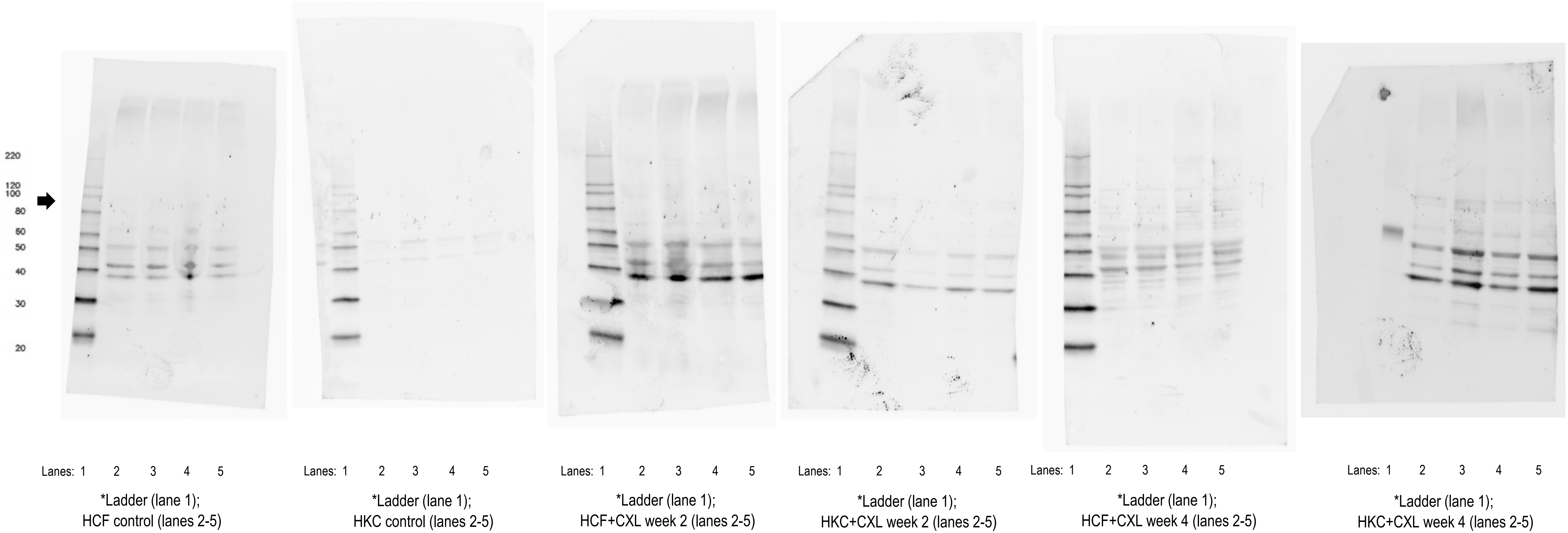

	Total Density					
PGC1	HCF control	HKC control	HCF+CXL 2 week	HKC+CXL 2 week	HCF+CXL 4 week	HKC+CXL 4 week
sample 1	2.09E+06	4.49E+05	3.03E+06	6.03E+06	2.22E+06	3.12E+06
sample 2	2.98E+06	1.78E+05	6.11E+06	1.10E+07	4.30E+06	1.30E+06
sample 3	1.97E+06	5.15E+05	1.87E+06	2.17E+06	2.67E+06	1.77E+06
sample 4	2.53E+06	5.43E+05	6.68E+06	7.58E+06	3.33E+06	4.13E+06

Supplemental Figure S6. Western Blot images and Total Density values of PGC1 protein expression in HCF and HKC 3D constructs following CXL treatment after 2 and 4 weeks. Constructs without treatment serve as controls. Each condition was repeated 4 times.

SRC molecular weight- 60 kDa; **MagicMark ladder molecular weights =** 20, 30, 40, 50, 60, 80, 100, 120, and 220 kDa

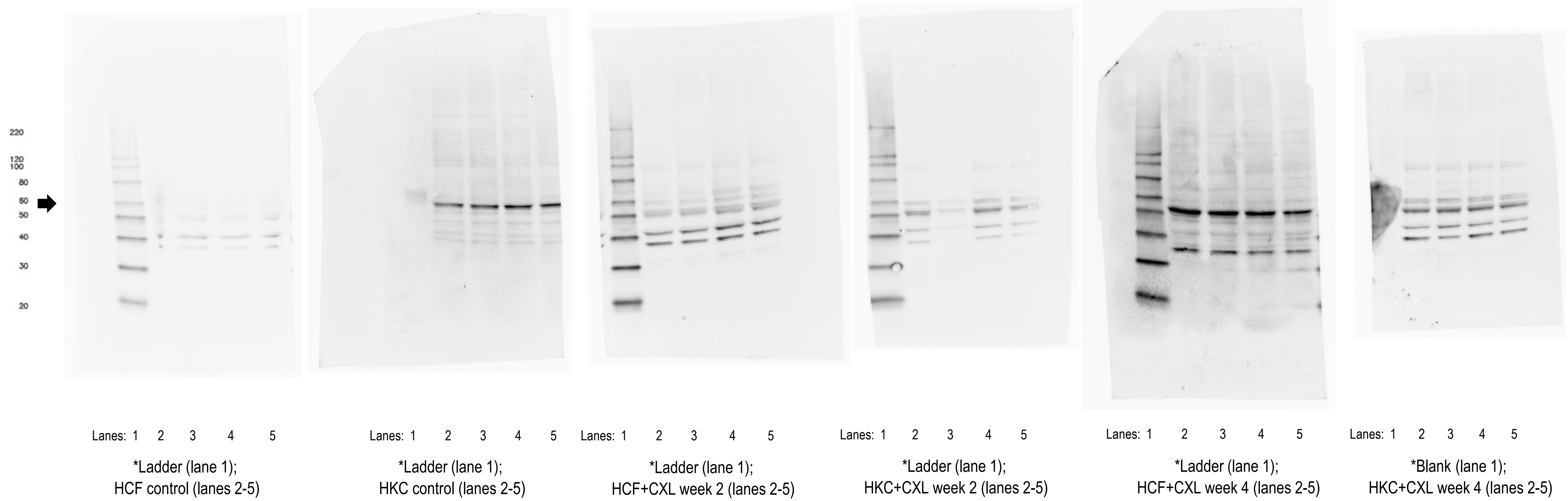

SRC	Total Density					
	HCF control	HKC control	HCF+CXL 2 week	HKC+CXL 2 week	HCF+CXL 4 week	HKC+CXL 4 week
sample 1	6.39E+03	4.69E+07	1.93E+04	8.73E+04	4.92E+06	3.02E+04
sample 2	4.48E+03	4.78E+07	2.70E+04	2.40E+04	7.50E+06	2.00E+04
sample 3	6.95E+03	6.45E+07	6.87E+04	1.37E+04	4.27E+06	2.97E+04
sample 4	1.23E+03	5.03E+07	6.88E+04	5.18E+04	4.13E+06	2.53E+04

Supplemental Figure S7. Western Blot images and Total Density values of SRC protein expression in HCF and HKC 3D constructs following CXL treatment after 2 and 4 weeks. Constructs without treatment serve as controls. Each condition was repeated 4 times.

CyclinD1 molecular weight- 34 kDa; **MagicMark ladder molecular weights** = 20, 30, 40, 50, 60, 80, 100, 120, and 220 kDa

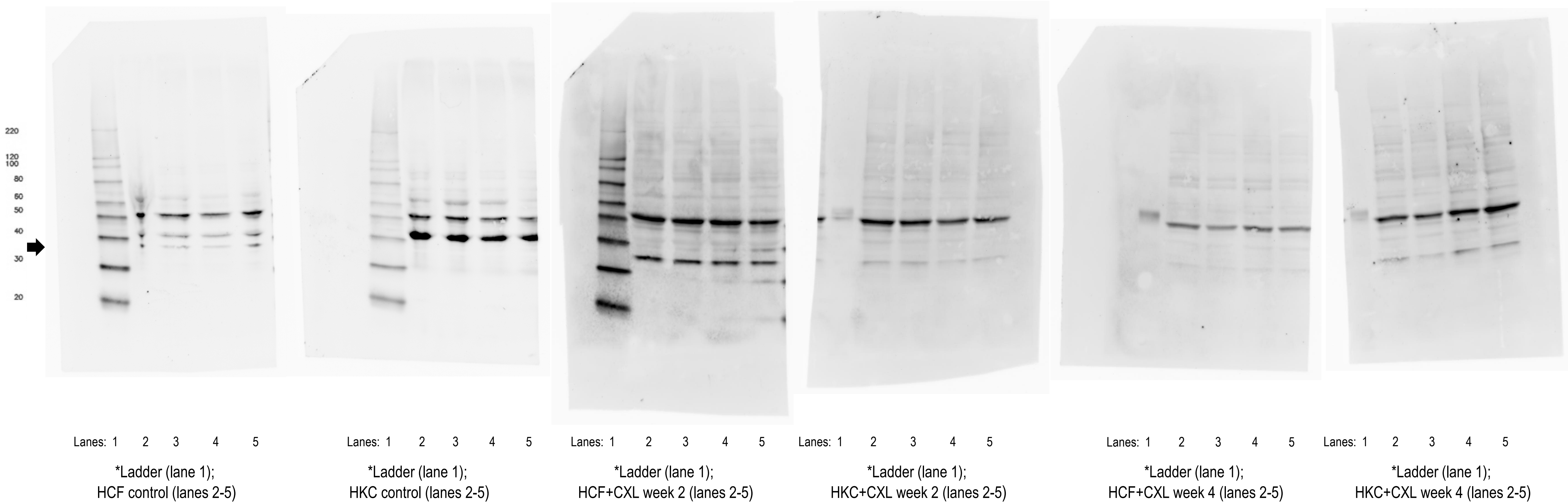

CyclinD1	Total Density					
	HCF control	HKC control	HCF+CXL 2 week	HKC+CXL 2 week	HCF+CXL 4 week	HKC+CXL 4 week
sample 1	6.39E+03	4.69E+07	1.93E+04	8.73E+04	4.92E+06	3.02E+04
sample 2	4.48E+03	4.78E+07	2.70E+04	2.40E+04	7.50E+06	2.00E+04
sample 3	6.95E+03	6.45E+07	6.87E+04	1.37E+04	4.27E+06	2.97E+04
sample 4	1.23E+03	5.03E+07	6.88E+04	5.18E+04	4.13E+06	2.53E+04

Supplemental Figure S8. Western Blot images and Total Density values of CyclinD1 protein expression in HCF and HKC 3D constructs following CXL treatment after 2 and 4 weeks. Constructs without treatment serve as controls. Each condition was repeated 4 times.
